# Supplementary material for: Structural and functional brain-wide alterations in A350V Iqsec2 mutant mice displaying autistic-like behavior
Source: Transl Psychiatry. 2021 Mar 22;11:181. doi: 10.1038/s41398-021-01289-8 (PMC7985214; doi:10.1038/s41398-021-01289-8)
Supplement: Supplementary file 1 — Supplemental Material [file 41398_2021_1289_MOESM1_ESM.docx]

**Supplementary Materials**

Structural and Functional Brain-wide Alterations in A350V *Iqsec2* Mutant Mice Displaying Autistic-like Behavior

Daniela Lichtman, Eyal Bergmann, Alexandra Kavushansky, Nadav Cohen, Nina S. Levy, Andrew P. Levy*, and Itamar Kahn*

Rappaport Faculty of Medicine, Technion–Israel Institute of Technology, Haifa 31096, Israel

**Quality assurance analysis**

To quantify the specificity of functional connectivity we conducted a quality analysis as suggested in a recent multi-center comparison of fcMRI data^1^. By comparing specific homotopic functional connectivity in the somatosensory cortex to unspecific connectivity between the somatosensory and anterior cingulate cortices in the individual wild-type mice included in the current study (**Fig. S1**). The analysis was done using seed-to-seed approach in which correlations between two regions were calculated and averaged across sessions for individual mice. All seeds (taken from supplementary data in ref. 2) had the same size to control for size-related signal-to-noise differences (**Fig. S1a**). First, a binary threshold was set by evaluating the distribution of the specific homotopic connectivity over multiple z(r) threshold^2^ (**Fig. S1b**). Next, the specific and unspecific functional connectivity of each animal was compared and classified according to a connectivity profile that was defined by the detected threshold (**Fig. S1c**). This analysis reveals a bias (i.e., values fall below the diagonal) toward the specific connection in all mice, as well as 9/13 mice which meet the criterion for definition of specific functional connectivity.

**Structure–function analysis**

The structure–function analysis used in this study was taken from Bergmann et al.^3^ and was further adjusted for intergroup comparison. First, we replicated the results shown in Bergmann et al.^3^ (see **Fig. 2**) for systems that are hierarchically organized (sensory networks, *n* = 24) relative to systems that are not predominantly hierarchical (association networks, *n* = 13). At the group level, seed-based statistical parametric maps were computed for WT mice (averaged across sessions) and then submitted to ROC analysis (**Fig. S2a**). Next, we modified the analysis to an individual level and replicated the sensory vs. association finding once again for analysis validation (**Fig. S2b**). At the individual level, we computed correlation maps (*z*(*r*)) of each individual animal (*n* =13), averaged all system-related seeds per animal, rather than the group average seed-based statistical parametric map (as was done above), and submitted it to the ROC analysis. For both levels, the location of the seeds (450 μm-diameter spheres) was defined at the center of the injection site. Anatomical projection volumes were taken from Oh et al.^4^ and compared to functional volume distributions over 158 statistical thresholds.

**References**

1 Grandjean J *et al.* Common functional networks in the mouse brain revealed by multi-centre resting-state fMRI analysis. *Neuroimage* **205**, 116278 (2020).

2 Bergmann E, Gofman X, Kavushansky A, Kahn I. Individual variability in functional connectivity architecture of the mouse brain. *Commun. Biol.* **3**, 738 (2020).

3 Bergmann E, Zur G, Bershadsky G, Kahn I. The organization of mouse and human cortico-hippocampal networks estimated by intrinsic functional connectivity. *Cereb. Cortex* **26**, 4497–4512 (2016).

4 Oh SW *et al.* A mesoscale connectome of the mouse brain. *Nature* **508**, 207–214 (2014).

**Supplementary Figures**

**
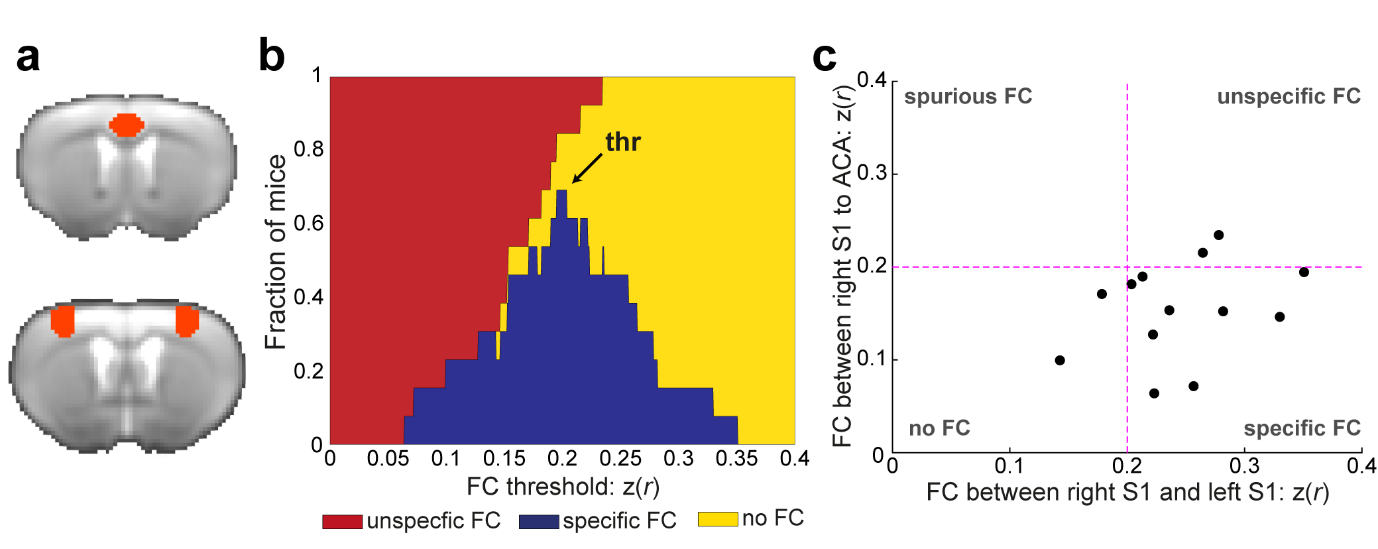
**

**Fig. S1. Functional connectivity specificity and sensitivity evaluation in WT mice.** **a** Location of seed regions in anterior cingulate cortex (ACA, *top*) and primary somatosensory cortex (S1, *bottom*). **b** Distribution of functional connectivity (FC) specificity as a function of z(r) threshold reveals a peak (thr) that serves as a binary threshold for quality evaluation. **c** Comparison between functional connectivity of the right S1 to either left S1 (specific ROI) or ACA (non-specific ROI) reveals bias toward the specific ROI in 9 out of 13 mice (Wilcoxon Signed-rank test, *p* < 0.001, Z = 3.18); z(r) threshold (magenta dashed lines) indicate classification of connectivity profile as “specific FC”, “unspecific FC”, “spurious FC” or “no FC” as described in ref. ^1^; z(r) values were averaged across sessions of each mouse.

**
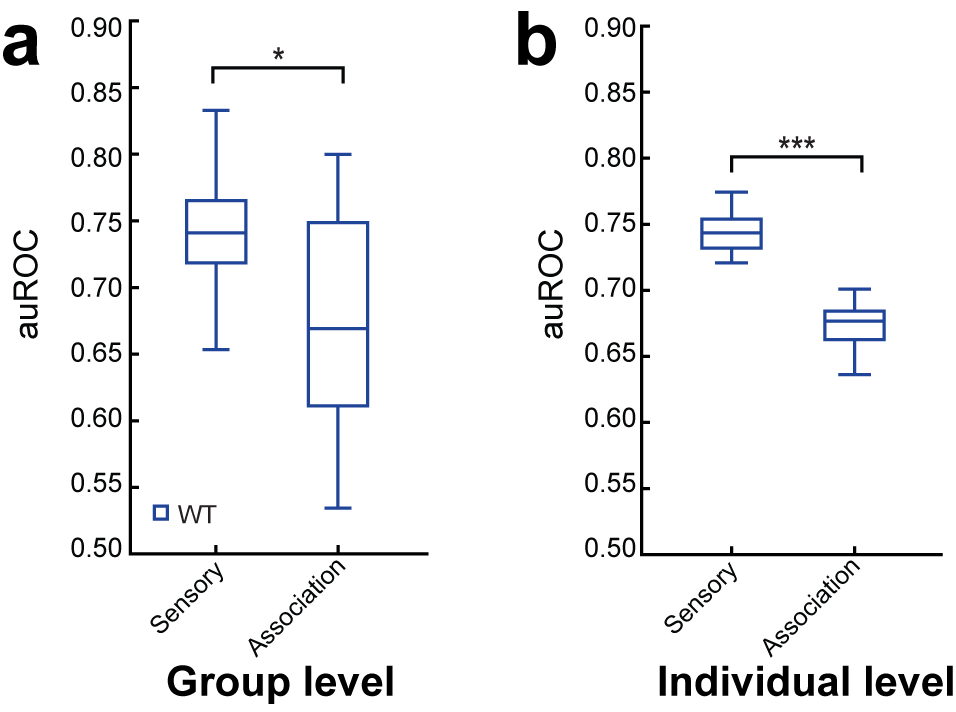
**

**Fig. S2. Structure–function relations of sensory and association systems in WT mice.** **a** The area under receiver operating characteristic (auROC) curve values demonstrate higher structure–function relation at the group level in sensory relative to association system (Mann–Whitney U Test; *n*_Sensory_ = 24, *n*_Association_ = 13, U = 84, *p* = 0.022). **b** auROC curve values demonstrate higher structure–function relation at the individual level in sensory relative to association system (Wilcoxon Signed-rank test; *n*_WT_ = 13 per system, *p* < 0.001, Z = 3.18). Boxplots represent the median (center line), interquartile range (box limits), extreme data points (whiskers) and outlier (circles). * *p* < 0.05, *** *p* < 0.001.
